# Computational Study of the Enhancement of Graphene Electrodes for Use in Li–Ion Batteries via Forming Superlattices with Transition Metal Dichalcogenides

**DOI:** 10.1021/acs.jpcc.3c06300

**Published:** 2024-01-04

**Authors:** Edward Allery David Baker, Conor Jason Price, Steven Paul Hepplestone

**Affiliations:** Department of Physics, University of Exeter, Exeter EX4 4QL, U.K.

## Abstract

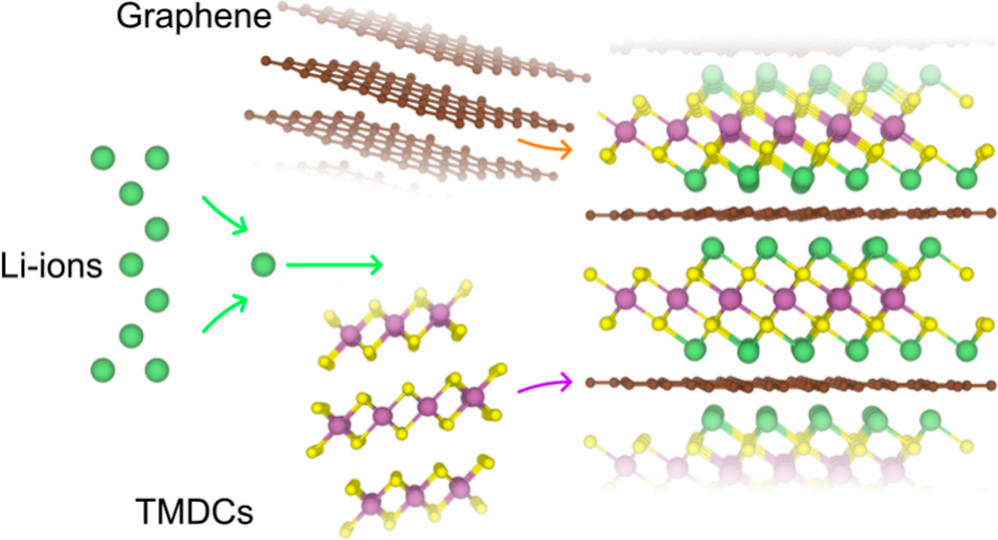

In our study, we examined nine transition metal dichalcogenide (TMDC)–graphene superlattices as potential Li–ion intercalation electrodes. We determined their voltages, with ScS_2_–graphene in T- and R-phases showing the highest at around 3 V, while the others ranged from 0 to 1.5 V. Most superlattices exhibited minimal volumetric expansion (5 to 10%), similar to NMC (8%), except for SnS_2_-T and NiS_2_-T, which expanded up to nearly 20%. We evaluated their capacities using a stability metric, *E*_IS_, and found that ScS_2_-T, ScS_2_-R, and TiS_2_-T could be intercalated up to two Li ions per MX_2_ unit without decomposing to Li_2_S, yielding capacities of 306.77 mA h/g for both ScS_2_ phases and 310.84 mA h/g for TiS_2_-T, roughly equivalent to LiC_2_. MoS_2_-T could accept Li up to a limit of *a* = 15/16 in Li_*a*_MoS_2_C_*b*_, corresponding to a capacity of 121.29 mA h/g (equivalent to LiC_4_). Examining the influence of graphene layers on MoS_2_-T, we observed a voltage decrease and an initial *E*_IS_ decrease before effectively flat lining, which is due to charge donation to the middle graphene layer, reducing the electron concentration near the TMDC layer. As graphene layers increased, overall volume expansion decreased with Li intercalation, which is attributed to the in-plane expansion changing. Our results underscore the potential of TMDC–graphene superlattices as Li–ion intercalation electrodes, offering low volumetric expansions, high capacities, and a wide voltage range. These superlattices all show an increase in the capacity of the graphene.

## Introduction

Li–ion-based batteries are the most widely used energy storage medium for portable electronic devices, seeing use in anything from phones to electric cars and most devices in between. The electrodes of a Li–ion battery determine their voltage and capacity, with many different types of materials having been used and investigated as Li–ion battery electrodes. These materials mainly consist of layered van der Waals structures following the discovery and use of TiS_2_ as a Li–ion intercalation electrode in the 1970s,^1−3^ which was followed by LiCoO_2_ in 1980.^4^ From this, many other layered materials were investigated, such as graphite,^5^ the nonlayered spinel structure LiMn_2_O_4_,^6,7^ and MoS_2_,^8−16^ before the currently commercially used NMC^17−19^ and NCA^20^ were discovered.

Many electrode materials share the layered structure of transition metal dichalcogenides (TMDCs), which are made of one part transition metal (M) and two parts chalcogen (S, Se, or Te, denoted as X) with the general formula MX_2_.^21^ Since the investigations into TiS_2_,^1−3^ TMDCs have remained a prevalent electrode,^22−25^ with sulfur-based TMDCs often being looked at over other TMDCs as they are lighter and have been shown to have higher capacities than selenium- and tellurium-based TMDCs.^26^ The large van der Waals gap TMDCs possess allows for the rapid insertion and extraction of intercalants, as demonstrated by systems such as VS_2_,^27^ while maintaining relatively low volume changes of 8% for materials such as NMC.^28^ This has led to more of these TMDCs to be investigated for use as Li–ion battery electrodes, such as WS_2_,^29^ NbSe_2_,^30^ ReSe_2_,^16^ and most recently, ScS_2_.^31^ Nb- and Ta-based materials^32^ have been shown to be intercalatable up to ratios of 1:1 Li/MX_2_, but their heavier masses result in lower theoretical capacities below 170 mA h/g. MoS_2_ is widely studied^8−16^ in the field of TMDCs and has been the subject of numerous investigations, demonstrating a capacity of 167 mA h/g but poor conductivity. ScS_2_ in particular has recently been suggested as a promising electrode,^31^ promising an ideal maximum cathode voltage of 4.5 V, a reversible capacity of 183 mA h/g, and a volumetric expansion of 7.5%. In addition, in spite of the ready conversion into Li_2_S and Sn, SnS_2_ also shows considerable promise as an electrode material.^33−40^

Recent studies have looked at improving many of the properties of TMDCs needed for their use as electrode materials with the aim of extending device operation, increasing the intercalant capacities, and improving conductivity. Morphology control^8,41,42^ and composite formation,^27,43−48^ particularly through the inclusion of graphitic carbon^49,50^ or other layered materials, have been used to improve electrical and ionic conductivity, provide mechanical support, and improve the resultant capacity. Carbon is also often used as the anode in Li–ion batteries and can be obtained in multiple forms. The most basic of these is graphite, which can be intercalated up to the LiC_6_ limit (equivalent to 339.18 mA h/g).^51^ Graphene has been shown to achieve a higher capacity, but this is often suspended in monolayers, which are unrealistic in a normal battery electrode.^52^

Constructing superlattices is an attractive approach for tailoring the properties of two-dimensional materials due to the comparative ease with which they can be made (such as via exfoliation^53^) and has been applied to TMDCs.^54−58^ Given the layered structure of many battery electrodes in use today, such as NMC, NCA, LiCoO_2_, and graphite, superlattices could be made from these to modify their voltages, capacities, thermal stability, and more in order to improve their overall performance. Some TMDC–graphene superlattices have already been investigated as intercalation electrodes, showing promise as anodes,^59−62^ with MoS_2_–graphene superlattices showing voltages of 1.5 V and conversion reactions to Li_2_S at 2.3 V.^61^ Experimental evaluations of the MoS_2_/graphene systems have indicated^62^ that such systems offer improvements in terms of diffusion pathways and could be used for dual Li–Mg systems.

In this article, we have investigated the effect that forming superlattices (alternating TMDC/graphene multilayer systems) with graphene has on a wide variety of sulfur-based TMDCs using density functional theory (DFT). We have calculated the voltages and capacities by looking at the thermodynamic relation between the TMDCs and byproducts that are often formed when these breakdown in the presence of Li, Li_2_X. We have also investigated the effect that additional graphene layers have on T-phase MoS_2_.

## Methods

### Density Functional Theory

First-principles DFT calculations were performed using the projector augmented wave (PAW)^63,64^ method implemented in the Vienna Ab initio Simulation Package (VASP).^65−68^ The calculations utilize Perdew–Burke–Ernzerhof electron exchange correlation functions.^69,70^ The plane wave energy cutoff and augmentation were both 500 eV, with a Γ-centered Monkhorst-pack grid^71^ of at least 3 × 3 × 3 was used for the supercells due to their size, denser grids were used for smaller supercells. van der Waals interactions were included using the DFT-D3 method of Grimme^72^ to account for the weak interactions between the 2D-layered materials. The structures were geometrically relaxed until the forces between the atoms were less than 0.01 eV/Å using a combination of the conjugate gradient algorithm^73^ and a quasi-Newtonian relaxation algorithm, RMM-DIIS.^74^ PAW pseudopotentials were used for core electrons, and the electrons that have been treated as valence are Mo 4d^5^5s^1^, W 5d^4^6s^2^, Sn 5s^2^5p^2^, Sc 3d^1^4s^2^, Ni 3d^8^4s^2^, Mn 3d^5^4s^2^, Ti 3d^2^4s^2^, S 3s^2^3p^4^, C 2s^2^2p^2^, and Li 1s^2^2s^1^.

**Figure 1:**
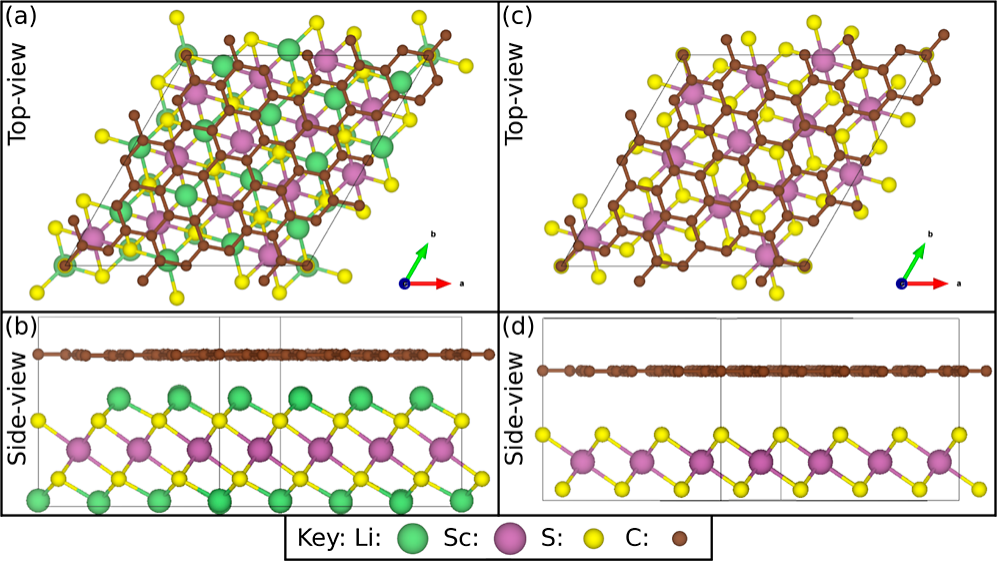
Schematic of ScS_2_ with graphene with Li from the top (a) and side (b) and without Li from the top (c) and side (d). This has 13 MX_2_ units and 56 carbon atoms.

In order to minimize the strain between the TMDCs and graphene, large supercells have been created with anywhere from 4 to 16 MX_2_ units, an example of which is shown in Figure 1. These supercells have been generated using the ARTEMIS^75^ package, which carries out a series of rotational matchings of two layers and produces a unit cell with minimal strain, alongside estimating the interlayer distances. The supercells were generated with and without Li (*a* = 0 and *a* = 2) for the 9 different TMDCs investigated, and these were then modified to get additional Li concentrations (*a* = 1, 15/16, 17/16). For such large supercells, the number of configurations for *a* = 1 would result in, for example, 60,000,000 calculations (for one system) and only provide an intermediary voltage. As such, we have focused on only the key points of intercalation (*a* = 0, 1, 2). For *a* = 1, we have chosen 2 configurations based on previous results for TMDCs,^26^ one with the Li filling every other interlayer region between graphene and the TMDC, and the second where the Li is evenly spread between the interlayer regions. The precise geometry of the relevant unit cells and how these additional concentrations were made are provided in the Supporting Information, Section S3. These were all calculated from a graphene supercell of the same size as in the TMDC–graphene superlattices, the exact sizes of these are given in Table 1, and a single unit cell of the TMDCs, the only exception to this is MnS_2_ T, for which we used a TMDC supercell of the same size, the reason for this is given in the Supporting Information, Section S3. Volume expansion with increasing Li content was calculated in the standard method,  where *V*_0_ is the volume of an unintercalated superlattice (*a* = 0).

**Table 1:** Ratios of MX_2_ to C Along With the Strain Associated with Each Layer for the 9 Supercells Generated Using the ARTEMIS^75^ Package and the Formation Energies per Unit Area^a^

TMDC	no. MX_2_	C/MX2 ratio (*b*)	strain on TMDC (%)	strain on graphene (%)	formation energy (meV/Å^2^)
MoS_2_ H	16	3.3750	0.98	–0.36	0.86
MoS_2_ T	16	3.3750	–0.59	–0.02	–8.95
WS_2_ H	16	3.3750	0.89	–0.35	0.043
SnS_2_ T	4	4.5000	0.65	–0.15	6.53
ScS_2_ R	13	4.3077	–3.44	0.018	13.38
ScS_2_ T	13	4.3077	–0.24	0.018	19.82
NiS_2_ T	13	3.8462	–3.44	–0.53	5.80
MnS_2_ T	7	3.7143	–4.74	–0.20	64.30
TiS_2_ T	16	3.8750	0.29	–0.27	11.69

a The strains are calculated for the TMDCs with no Li compared with their superlattices with no Li (*a* = 0).

### Voltages and Stability

The voltages, *V*, of these superlattices at different levels of Li intercalation can be expressed as
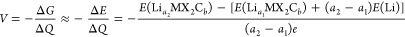
1where Δ*G* is the change in Gibbs free energy, the total Li content *a*_2_ > *a*_1_, *E*(Li_*a*_MX_2_C_*b*_) is the energy of an MX_2_–graphene superlattice with *a* Li and *b* carbon per MX_2_ unit, and *E*(Li) is the energy of Li in its bulk form. The Gibbs free energy can be approximated as the internal energy, as the pressure–volume and vibrational entropy contributions are known to be negligible in TMDCs^76^ and graphite/graphene.^77^

The thermodynamic stability of these superlattices was assessed by looking at the favorability of the formation of the secondary product Li_2_X. In Li–ion batteries, the formation of secondary products like Li_2_X indicates a loss of the desired layered structure, leading to a loss in capacity. We can determine the maximum Li intercalation limit by finding a region in phase space where Li_2_X is less favorable than an intercalated superlattice, where an unintercalated structure is less favorable, and where the elemental bulks are less favorable. These limits are expressed as
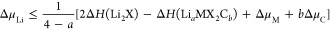
2
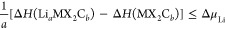
3
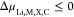
4where Δ*H*(A) is the enthalpy of formation of compound A with respect to the bulk constituents and Δμ_A_ is given by Δμ_A_ = μ_A_ – μ_A_^0^, μ_A_ being the chemical potential of species A when in Li_*a*_MX_2_C_*b*_, with A = Li, M, X, and C. If we consider the maximum difference in Δμ_Li_ in eqs 2 and 3 when Δμ_M_ = Δμ_C_ = 0, we can qualitatively determine if a region of stability exists for Li_*a*_MX_2_C_*b*_, we define this quantity as *E*_IS_, and it is given by
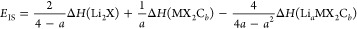
5

A positive *E*_IS_ means that Li_*a*_MX_2_C_*b*_ is thermodynamically favorable, and a negative *E*_IS_ means that Li intercalated to this capacity is not stable and will result in the formation of Li_2_S. Hence, determining the limit of intercalation, *a*, for when *E*_IS_ = 0, determines the maximum amount of Li that can be intercalated and therefore the capacity. For these superlattices, we have also considered the formation of LiC_6_ from the lithiated superlattices (*a* > 0) and show that this is always unfavorable. The origin of the limits that *E*_IS_ is derived from and the results looking at the formation of LiC_6_ can be found in Supporting Information, Sections S1 and S2.

## Results and Discussion

### General Properties

To establish the viability of these TMDC–graphene superlattices for the intercalation of Li, we first need to examine the resultant strains and formation energies. The exact number of MX_2_ units and the carbon to MX_2_ ratio (*b*) is given in Table 1, along with the strain associated with each layer and the formation energy per unit area. These are calculated for the TMDCs with no Li compared with their respective superlattices with no Li (*a* = 0). Details of this are given in the Supporting Information, Section S3. As can be seen, the strains are all less than ±0.6% for graphene and less than ±5% for the TMDCs. All of the formation energies for these structures are less than ±0.02 eV/Å^2^, with the exception of MnS_2_. However, in all cases, these supercells are both energetically viable and have sufficiently low strains to not dramatically affect the resultant properties. In general, the graphene shows minimal strain, whereas the TMDCs are more strained; however, these are lower than the expansion that these materials undergo due to Li intercalation. It is of note that, in all cases, the inclusion of graphene has made these superlattices both conductive, which is required for them to be used as electrodes, and decreased the diffusion barriers (see Supporting Information).

### Voltages

**Figure 2:**
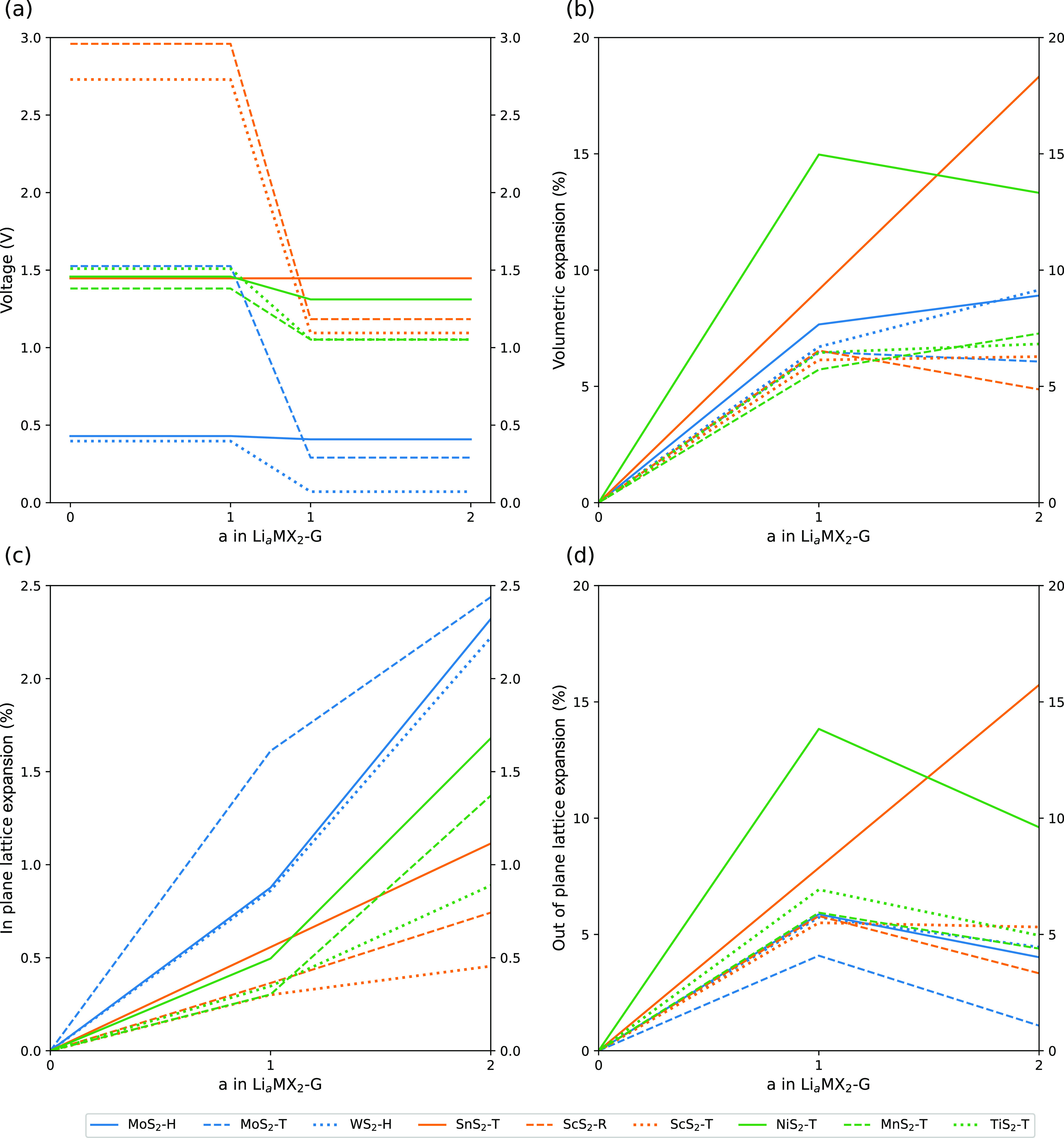
Open circuit voltages (a), volumetric expansions (b), in-plane lattice expansions (c), and out-of-plane lattice expansions (d) for the TMDC–graphene superlattices as *a* increase in Li_*a*_MX_2_-G.

The voltage is one of the most fundamental properties of an electrode and is used to determine if it is considered an anode or a cathode. Anodes normally have voltages lower than 2 V vs Li/Li^+^, ideally between 0.5 and 1.5 V, cathodes normally have voltages higher than 3 V, ideally between 3 and 4.5 V.^78^Figure 2a shows how the voltage of the TMDC–graphene superlattices varies as *a* in Li_*a*_MX_2_C_*b*_ is increased, calculated using eq 1.

Most of the TMDC–graphene superlattices display voltages that lie in the anode range. MoS_2_-H, MoS_2_-T, WS_2_-H, SnS_2_-T, NiS_2_-T, MnS_2_-T, and TiS_2_-T all have voltages between ≈1.5 and 0 V, meaning that these would be suitable as anodes. Despite how similar MoS_2_-H and MoS_2_-T are in composition, the change in TMDC phase leads to MoS_2_-T having a far higher voltage for *a* = 0 → 1, this may be caused by the large rearrangement that this system has undergone, having changed from T-phase to T′-phase (a distortion of T-phase with alternating Mo–Mo distances) and back to T-phase for *a* = 0 → 1 → 2. MoS_2_–H has a very flat voltage, which is far more preferable when looking for a Li intercalation battery electrode. SnS_2_-T has a flat voltage from *a* = 0 to *a* = 2, as neither of the *a* = 1 configurations was more favorable than a combination of the *a* = 0 and *a* = 2 structures. Our results for MoS_2_-T with graphene agree with those of the experiment, where a voltage of ≈1.5 V has been seen, as well as irreversible conversion reactions occurring above this voltage.^61^ We note that WS_2_, ScS_2_-R, and ScS_2_-T all show a significant drop in their voltages.

ScS_2_-R and ScS_2_-T both start with much higher voltages, closer to 3 V for *a* = 0 → 1, before dropping down to an anode-like voltage, around 1.15 V for *a* = 1 → 2. This puts them in an odd situation where they are not quite high enough to be considered a cathode but start too high to be considered an anode for their whole intercalation range at this ratio of MX_2_ to carbon. This suggests that ScS_2_-based cathodes should have low levels of carbon such that their voltages are not as significantly decreased.

Comparison of the TMDC–graphene voltages with those of their bulk TMDCs shows that the voltage is generally decreased. The details of this are shown in Supporting Information, Section S4. The lowest decrease was shown when mixing WS_2_–H with graphene, which was only decreased by 1.4%. The highest decrease in voltage was the mixing of MoS_2_–H with graphene, which showed a decrease in voltage of 0.72 V without graphene to 0.43 V with graphene. In general, our results show that the addition of graphene decreases the voltages of the TMDCs, with values ranging from 1.44 to 40.38% in the range *a* = 0 → 1. Clearly, the inclusion of graphene increases the effectiveness of these materials as anodes but is detrimental to the performance of cathodes.

### Volumetric Expansion

When investigating Li–ion intercalation electrodes, it is important to look at how the volume of these materials changes during cycling, as this can be a cause of degradation of these materials that leads to a loss of usable capacity. Figure 2b shows how the volume of the TMDC–graphene superlattices varies as *a* in Li_*a*_MX_2_C_*b*_ is increased. As expected, for all superlattices investigated, we can see a general increase in volume when Li is intercalated. The superlattices at *a* = 1 and *a* = 2 all show smaller volumetric expansions to their respective bulk TMDCs at *a* = 1, with the exception of WS_2_-H and SnS_2_-T, which expand more at *a* = 2, these are given in the Supporting Information, Section S5. Bulk MnS_2_-T has actually shrunk when Li is intercalated into it; this is due to the aligned spins on the manganese atoms going from 3 up with no Li to 2 up with Li; the aligned spins repulse each other, leading to a decrease in volume. This is not observed in our MnS_2_-T superlattice, as the manganese atoms are more spatially separated due to the inclusion of graphene between the TMDC layers.

The observed expansions of the superlattices can be split into three groups, showing slightly different trends. MoS_2_-H, WS_2_-H, ScS_2_-T, MnS_2_-T, and TiS_2_-T have a large increase in volume for *a* = 0 → 1 with a much smaller increase in volume for *a* = 1 → 2, this is what we expect to happen for the vast majority of the investigated superlattices as Li prefers to be split between both sides of the TMDC layer instead of all on one side, meaning that both gaps between the TMDC and graphene layers are spread apart by Li. Although Li ScS_2_-T prefers to all be on one side when *a* = 1, breaking this “trend”. SnS_2_-T has a constant expansion from *a* = 0 → 2 due to the *a* = 1 configuration not being favorable. MoS_2_-T, ScS_2_-R, and NiS_2_-T all show an odd behavior of contracting when going from *a* = 1 → 2, this is also observed experimentally for materials such as NMC, which has the largest out-of-plane lattice constant near 50% Li content.^28^

From the lattice constant expansions shown in Figure 2c,d, we can see that the expansion of the out-of-plane lattice is the biggest contributor to the volumetric expansion. This is due to the van der Waals gaps between the layers being forced apart by Li as it is intercalated; this is seen in many other intercalation electrodes.^28^ We see volumetric expansions in the range of 5 to 10% for all materials except for SnS_2_-T and NiS_2_-T. This level of expansion is comparable to that seen in materials used in commercial batteries, such as NMC, with an expansion of 8%.^28^ SnS_2_-T and NiS_2_-T both undergo expansion in the range of 10 to 20%, which could lead to the significant formation of cracks during cycling, which accelerate degradation and limit capacity.^79^

### Chemical Stability and Capacity

**Figure 3:**
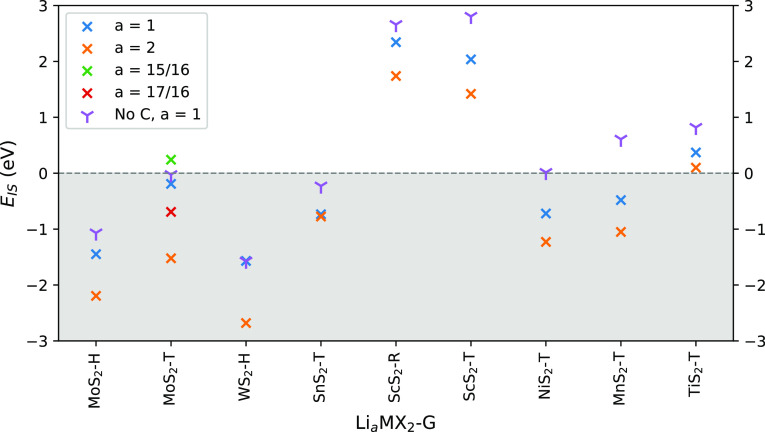
*E*_IS_ for the TMDC–graphene superlattices as the intercalation level, *a*, increases in Li_*a*_MX_2_C_*b*_ for *a* = 1 and *a* = 2. Value for *E*_IS_ has been included for these without graphene as well. For MoS_2_, extra points have been included for *a* = 15/16 and *a* = 17/16.

We can determine the maximum amount of Li that the TMDC–graphene superlattices can accommodate by determining when *E*_IS_ ≈ 0 by using eq 5. Figure 3 shows how *E*_IS_ varies as the level of intercalated Li, *a*, is increased. In these structures, we investigate strictly one layer of carbon with one layer of TMDC. For all the systems that have positive *E*_IS_, a similar or better Li to carbon ratio than that of graphene on (LiC_6_) its own is achieved. This indicates that the capacity of the carbon has been increased. The superlattices that show this improvement involve the TMDCs ScS_2_-R, ScS_2_-T, TiS_2_-T, and MoS_2_-T. Conversely, the remaining TMDCs are considered to readily decompose into Li_2_S at *a* = 1. These may still be able to intercalate Li without undergoing conversion; however, intercalation for *a* < 1 will lead to low capacities that are not suitable for electrodes.

To further explore the limits of mixing these systems, we expand on the results for MoS_2_-T with graphene. For MoS_2_-T at *a* = 1 and *a* = 2, the *E*_IS_ is negative, meaning that it is unfavorable to intercalate this TMDC even to just one Li per unit. However, the value of *E*_IS_ at *a* = 1 was very low, equal to −0.191 eV. We have considered Li concentrations of *a* = 15/16 and *a* = 17/16 (±1 Li compared to *a* = 1). From this, we can confirm that *E*_IS_ does become positive for *a* = 15/16, equal to 0.242 eV, meaning that MoS_2_-T with graphene is able to be intercalated and has a capacity equal to 121.29 mA h/g. If we look at the graphene layer, this is roughly equivalent to the limit of LiC_4_. We can also see that the value of *E*_IS_ at *a* = 17/16 is between the values of *E*_IS_ at *a* = 1 and *a* = 2. Thus, we can state that the MoS_2_–graphene boundary shows a small increase in capacity compared to the pure graphene region and a slight decrease in performance when compared to pure MoS_2_. This result agrees with Larson et al.^59^ who showed, for Li_*a*_MoS_2_C_3.125_ (50C: 16 MoS_2_), that the limit of intercalation is *a* ≈ 1.

ScS_2_-R, ScS_2_-T, and TiS_2_-T are all resistant to the formation of Li_2_S up to an intercalation of *a* = 2, meaning that these can be intercalated up to at least 2 Li per MX_2_ unit without decomposing. When ScS_2_-R and ScS_2_-T are compared to graphene, this is almost approaching LiC_2_, a large improvement over the LiC_6_ limit of graphene. When TiS_2_ is compared to graphene, it is equivalent to going slightly beyond LiC_2_. Both ScS_2_-R and ScS_2_-T have a capacity of 306.77 mA h/g, and TiS_2_ has a capacity of 310.84 mA h/g.

Comparing the *E*_IS_ of the TMDC–graphene superlattices to their respective TMDCs at *a* = 1, we can see that the addition of graphene has decreased the stability of the TMDC against intercalation for all systems with the exception of WS_2_-H. This is shown by a general decrease in *E*_IS_, we find that this decrease compared to the bulk TMDC ranges from 1.09 to 0.17 eV, excluding WS_2_-H, which increases by 0.01 eV.

### Changing the Ratio of Carbon to TMDC

**Figure 4:**
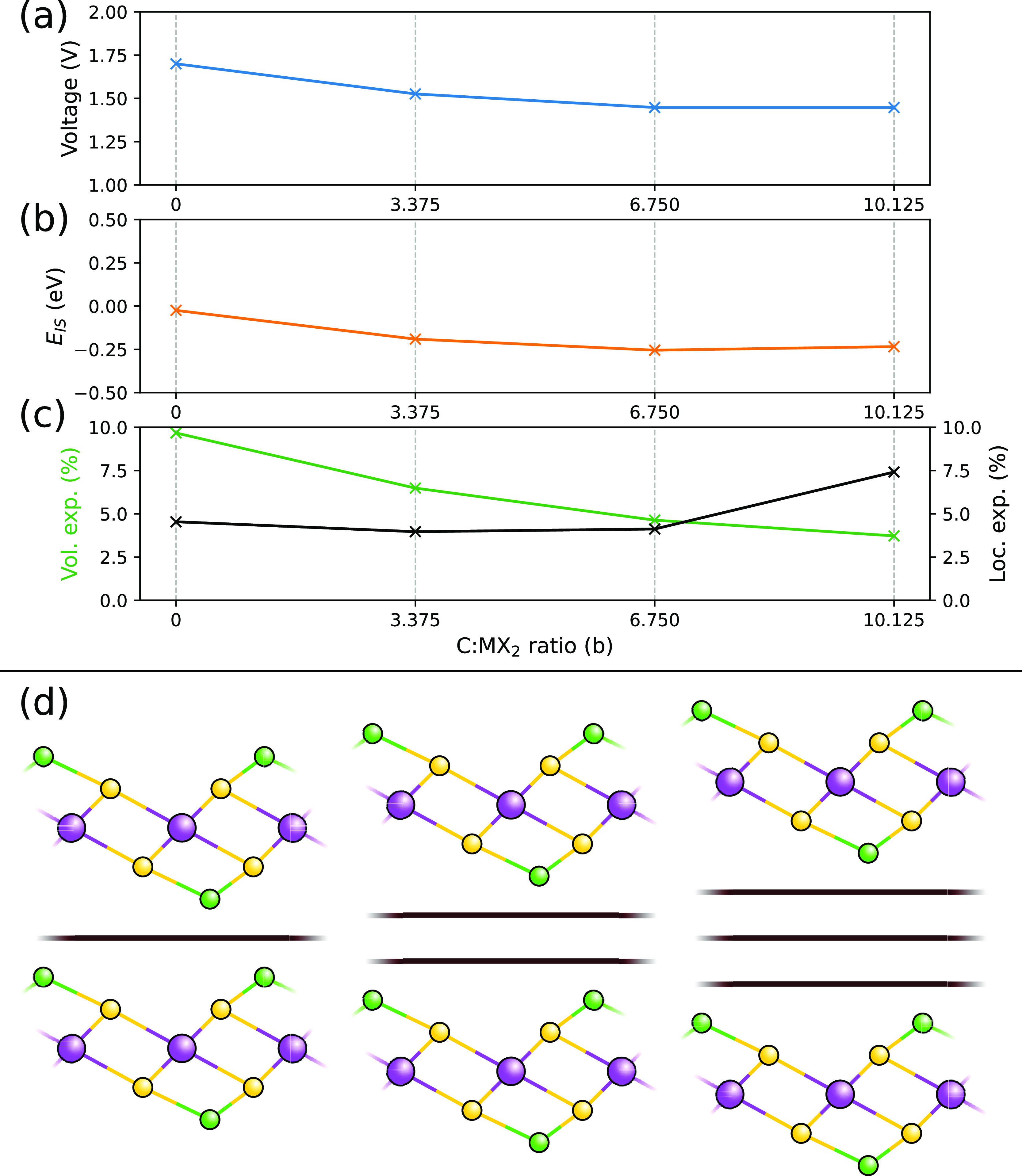
(a) Open circuit voltage and (b) *E*_IS_ for MoS_2_-T as the number of layers of graphene is increased for a Li content of *a* = 0 and *a* = 1. (c) Shows both the total volumetric expansion in green and the local expansion in the *z*-axis in black. For the superlattices, this is measured from the graphene layer below the TMDC to the graphene layer above; for the TMDC on its own, we have used the distance between the closest sulfur atoms in the two neighboring TMDC layers, (d) is a schematic showing how additional carbon has been added to these systems as additional layers.

In order to investigate the effect of graphene further, we looked at what happens when the ratio of graphene to MoS_2_-T is increased. This system is of particular relevance as MoS_2_ does not conduct without an additive, such as hard carbons. We have used MoS_2_-T at Li contents of *a* = 0 and *a* = 1 for this, varying the number of graphene layers between 0 and 3, which is equivalent to *b* = 0, 3.375, 6.750, and 10.125 (intermediate cases are discussed in the Supporting Information). For all cases where *b* ≠ 0, the same local structure of MoS_2_-T and Li has been used, as was found for *a* = 1 and *b* = 3.375, no additional Li is added as the amount of carbon is increased, a schematic of this is shown in Figure 4c. From these results, we can assess the effect that more carbon has on the local stability, volume expansion, and voltage of the MoS_2_-T layer. The voltage, *E*_IS_ and volumetric, and local expansions are shown in Figure 4 and are given in the Supporting Information, Section S6.

Our results show that the voltage (from *a* = 0 to *a* = 1) decreases as the amount of carbon, *b*, is increased. As we have limited our search to just the interface region, this suggests that a truly mixed MoS_2_/hard carbon system (which has bulk regions of both materials) will observe three peaks in its voltage behavior. The first peak will occur at around 1.7 V and be associated with bulk MoS_2_ intercalation. The second peak will occur at 1.5 and be associated with the interface, and the final much lower peak will be associated with hard carbon (typically of the order of 0.5–1 V depending on the carbon). This has been observed in the experiment by Wenelska et al.^61^ where the interface peak occurs as a shoulder to the main bulk MoS_2_ peak with an additional peak at 2.3 V, which corresponds to the breakdown into Li_2_S.

Looking at the overall expansion of the various carbon superlattices, we can see that the volume expansion decreases with the amount of graphene layers. Given that we are not adding any additional Li as we increase the number of graphene layers, we need to instead look at how the local environment around the TMDC changes. In order to understand this local environment better, we have also included the local change in the out-of-plane direction, which is measured from the graphene layer just below the TMDC to the graphene layer just above. From this, we can actually see that the amount of out-of-plane space occupied by the TMDC and Li increases as the number of graphene layers increases. This indicates that the overall decrease in volume expansion as the amount of carbon increases is due to changes in the in-plane lattice expansion.

Our results also show that the local stability of the MoS_2_-T layer is also decreasing compared to bulk MoS_2_-T, with *E*_IS_ dropping as the amount of carbon *b* is increased. We can also see that both the voltage and *E*_IS_ change in unison, decreasing by roughly the same amount as *b* is increased. Initially, we see a decrease in *E*_IS_. This is due to the donated charge from the Li mainly being absorbed by the TMDC, with the graphene not absorbing significant amounts. However, we do see a slight increase in *E*_IS_ going from *b* = 6.750 to 10.125, where Bader charge analysis^80^ indicates that some of the donated charge is now spread to the third graphite layer, which reduces the total charge in the vicinity of the Li-TMDC region and could be responsible for this improvement in stability, details of this are provided in the Supporting Information, Section S6.

## Conclusions

In this work, we have investigated the performance of 9 different TMDC–graphene superlattices for their potential use as Li–ion intercalation electrodes. We have calculated their voltages, finding that ScS_2_–graphene in both the T- and R-phases possesses voltages nearing 3 V, while the other 7 TMDC–graphene superlattices are between 0 and 1.5 V. The vast majority of these superlattices also show very little volumetric expansion in the range of 5–10%, similar to that of NMC at 8%; the only exceptions to this are SnS_2_-T and NiS_2_-T, which expanded up to nearly 20%.

Looking at the breakdown of these superlattices into Li_2_S, LiC_6_, and their constituent transition metals, we assess their capacities using a metric of stability, *E*_IS_. From all of the superlattices investigated, we found that ScS_2_ in both T- and R-phases and TiS_2_-T are able to be intercalated up to two Li ions per MX_2_ unit (Li_2_MX_2_C_*b*_), leading to large capacities of 306.77 mA h/g for both ScS_2_ phases and 310.84 mA h/g for TiS_2_-T, which were both roughly equivalent to a limit of LiC_2_. MoS_2_-T was also found to be able to accept Li up to a limit of *a* = 15/16 in Li_*a*_MoS_2_C_*b*_, agreeing with results seen in other studies.^59^ This corresponds to a capacity of 121.29 mA h/g, which is equivalent to a limit of LiC_4_.

To further explore the effects of graphene in these superlattices, we investigated what would happen to MoS_2_-T as additional layers of graphene were added. This showed that adding more layers of graphene decreased the voltage, while our metric of stability, *E*_IS_, initially decreased before effectively flatlining. A Bader charge analysis revealed that this may be due to charge being donated to the middle graphene layer, reducing the amount of electrons near the TMDC layer. The overall volume expansion of these superlattices decreases with Li intercalation as the number of graphene layers increases, while the local expansion around the TMDC layer increases.

Our results highlight the effects of forming superlattices with TMDCs and graphene for use as Li–ion intercalation electrodes, with low volumetric expansions and high capacities with a wide range of voltages. All of the superlattices investigated became conductive due to the addition of graphene.

## Supporting Information Available

Derivation of *E*_IS_, formation of LiC_6_, lattice constants, stresses, strains, voltages, volumetric expansions, effects of additional graphene and MoS_2_, charge analysis, diffusion barriers, and AIMD stability (PDF)
