# Supplementary material for: Computational Study of the Enhancement of Graphene Electrodes for Use in Li–Ion Batteries via Forming Superlattices with Transition Metal Dichalcogenides
Source: J Phys Chem C Nanomater Interfaces. 2024 Jan 4;128(2):723–31. doi: 10.1021/acs.jpcc.3c06300 (PMC10801692; doi:10.1021/acs.jpcc.3c06300)
Supplement: Supplementary file 1 — jp3c06300_si_001.pdf [file jp3c06300_si_001.pdf]

Supporting Information
A Computational Study of the Enhancement of Graphene Electrodes for use in Li-Ion Batteries via Forming Superlattices with Transition Metal Dichalcogenides

Edward Allery David Baker,* Conor Jason Price, and Steven Paul Hepplestone*

Department of Physics, University of Exeter, Exeter, EX4 4QL

E-mail: eb531@exeter.ac.uk; S.P.Hepplestone@exeter.ac.uk

Derivation of E_{IS}

The stability of these TMDC-graphene superlattices has been characterised by the formation of Li_2S. When this compound forms in TMDCs it means that a irreversible degradation has occurred, removing both lithium and the chalcogen, which collapses the van der Waals layers that allow for Li-ion intercalation. By assessing the relative stability of these TMDC-graphene superlattices against Li_2S we can construct a phase diagram that indicates when one is more favourable than the other. First we define the enthalpy of formation of relevant products

$$\Delta H(\mathrm{Li_aMS_2C_b}) = E(\mathrm{Li_aMS_2C_b}) - \left[a\mu^0_{Li} + \mu^0_M + 2\mu^0_S + b\mu^0_C\right], \tag{S1}$$

$$\Delta H(\mathrm{MS_2C_b}) = E(\mathrm{MS_2C_b}) - \left[\mu_M^0 + 2a\mu_S^0 + b\mu_C^0\right], \tag{S2}$$

$$\Delta H(\mathrm{Li_2S}) = E(\mathrm{Li_2S}) - \left[2\mu_{Li}^0 + \mu_S^0\right], \tag{S3}$$

where $\Delta H(\mathrm{A})$ is the enthalpy of formation of compound A, $E(\mathrm{A})$ is the energy of compound A and $\mu_B^0 = E(\mathrm{B})$ is the chemical potential of the element B when it is in its elemental bulk structure and M represents transition metal. Thermodynamic equilibrium requires that

$$\Delta H(\mathrm{Li_aMS_2C_b}) = a\Delta\mu_{Li} + \Delta\mu_M + 2\Delta\mu_S + b\Delta\mu_C, \tag{S4}$$

where $\Delta\mu_B = \mu_B - \mu_B^0$ with μ_B being the chemical potential of the element B in $\mathrm{Li_aMS_2C_b}$. This states that the energy of the intercalated superlattice is the sum of the chemical potentials of its constituent atoms. This can be restated as

$$\Delta\mu_S + \frac{b}{2}\Delta\mu_C = \frac{1}{2}[\Delta H(\mathrm{Li_aMS_2C_b}) - a\Delta\mu_{Li} - \Delta\mu_M]. \tag{S5}$$

We also require that $\mathrm{MS_2C_b}$, $\mathrm{Li_2S}$ and the bulk forms of the constituent elements do not form, thus

$$\Delta\mu_M + 2\Delta\mu_S + b\Delta\mu_C \leq \Delta H(\mathrm{MS_2C_b}) \tag{S6}$$

$$2\Delta\mu_{Li} + \Delta\mu_S \leq \Delta H(\mathrm{Li_2S}), \tag{S7}$$

$$\Delta\mu_{Li,M,S,C} \leq 0. \tag{S8}$$

Substituting (S5) into (S6) and rearranging

$$\frac{1}{a}[\Delta H(\mathrm{Li_aMS_2C_b}) - \Delta H(\mathrm{MS_2C_b})] \leq \Delta\mu_{Li}, \tag{S9}$$

which is our first thermodynamic limit on the chemical potential of lithium that determines when lithium intercalated into $\mathrm{MS_2C_b}$ is more favourable than $\mathrm{MS_2C_b}$ and bulk lithium. Substituting (S5) into (S7) provides

$$\Delta\mu_{Li} \leq \frac{1}{4-a}[2\Delta H(\mathrm{Li_2S}) - \Delta H(\mathrm{Li_aMS_2C_b}) + \Delta\mu_M + b\Delta\mu_C], \tag{S10}$$

which is our second thermodynamic limit on the chemical potential of lithium that determines when $\mathrm{Li_2S}$ does not form. Together these describe the boundary conditions on the chemical potential of lithium based on the formation energies and chemical potentials of relevant products and elements. Given that we have removed all dependence on the chemical potential of the chalgogen (sulphur for all TMDCs investigated), these are dependent only on $\Delta\mu_{Li}$, $\Delta\mu_M$ and $\Delta\mu_C$.

If we consider the relative change in $\Delta\mu_{Li}$ between these two boundaries, Equations(S9) and (S10), at $\Delta\mu_M = \Delta\mu_C = 0$, we can quantise a region of stability using a single value E_{IS}. We define this quantity such that a positive value means that there is a region of stability and a negative means there is not, E_{IS} is thus defined as

$$\begin{aligned} E_{IS} = & \frac{2}{4-a}\Delta H(\mathrm{Li_2S}) + \frac{1}{a}\Delta H(\mathrm{MS_2C_b}) \\ & - \frac{4}{4a-a^2}\Delta H(\mathrm{Li_aMS_2C_b}). \end{aligned} \tag{S11}$$

This can also be used for TMDCs without graphene by simply setting $b = 0$.

The values of E_{IS} for the TMDC-graphene superlattices at $a = 1$ and $a = 2$ and the TMDCs without graphene at $a = 1$ are given in Table S S1.

Table S1: The values of E_{IS} for the TMDC-graphene superlattices at $a = 1$ and $a = 2$ and the bulk TMDCs at $a = 1$.

	E_{IS} (eV)		
	TMDC-graphene		TMDC
TMDC	a = 1	a = 2	a = 1
MoS_2-H	-1.4486	-2.1940	-1.0687
MoS_2-T	-0.1906	-1.5215	-0.0248
WS_2-H	-1.5692	-2.6800	-1.5742
SnS_2-T	-0.7345	-0.7748	-0.2290
ScS_2-R	2.3434	1.7392	2.6594
ScS_2-T	2.0365	1.4198	2.8057
NiS_2-T	-0.7209	-1.2284	0.0136
MnS_2-T	-0.4807	-1.0503	0.6082
TiS_2-T	0.3709	0.0994	1.1652

Formation of LiC_6

In addition to the formation of LiS_2, we have also considered the formation of LiC_6 from the intercalated superlattices. We can state this as

$$\mathrm{Li_aMS_2C_b} \longrightarrow (a-2\beta)\mathrm{LiC_6} + \beta\mathrm{Li_2S} + \beta\mathrm{M} + (1-\beta)\mathrm{MS_2} + (12\beta - 6a + b)\mathrm{C}, \tag{S12}$$

which has limits of

$$\beta \leq \frac{a}{2}, \tag{S13}$$

$$\beta \geq \frac{6a-b}{12}. \tag{S14}$$

These represent when there is no LiC_6 formed in the limit there is no additional carbon. Between these limits we found that $Li_aMS_2C_b$ was more favourable for all investigated TMDC-G superlattices. Tables SS2, SS3 and SS4 show the formation energy of $Li_aMS_2C_b$ from all compounds on the right hand side of equation S12. A negative formation energy shows that the formation of $Li_aMS_2C_b$ is more favourable, so LiC_6 won't form. All tables show negative

formation energies indicating LiC_6 will not form.

Table S2: The energetic costs of forming $Li_{a=1}MS2C_b$ from LiC_6 and various other compounds, based upon equation S12.

	Lower Limit		Upper Limit	
TMDC	Form. En. (eV)	β	Form. En. (eV)	β
MoS_2-H	-0.8749	0.2188	-1.69	0.5
MoS_2-T	-1.9736	0.2188	-2.5676	0.5
WS_2-H	-0.8029	0.2188	-1.5488	0.5
SnS_2-T	-1.188	0.125	-1.8443	0.5
ScS_2-R	-3.1184	0.141	-4.7093	0.5
ScS_2-T	-2.7626	0.141	-4.4059	0.5
NiS_2-T	-1.3646	0.1795	-1.7052	0.5
MnS_2-T	-1.743	0.1905	-2.2955	0.5
TiS_2-T	-1.8582	0.1771	-3.2318	0.5

Table S3: The energetic costs of forming $Li_{a=2}MS2C_b$ from LiC_6 and various other compounds, based upon equation S12.

	Lower Limit		Upper Limit	
TMDC	Form. En. (eV)	β	Form. En. (eV)	β
MoS_2-H	-2.5199	0.7188	-3.3351	1.0
MoS_2-T	-3.1075	0.7188	-3.7015	1.0
WS_2-H	-1.9872	0.7188	-2.7332	1.0
SnS_2-T	-3.461	0.625	-4.1173	1.0
ScS_2-R	-6.3053	0.641	-7.8962	1.0
ScS_2-T	-5.9334	0.641	-7.5767	1.0
NiS_2-T	-2.9944	0.6795	-3.335	1.0
MnS_2-T	-3.4747	0.6905	-4.0272	1.0
TiS_2-T	-4.8247	0.6771	-6.1982	1.0

Supercells

The in-plane and out of plane lattice constants for the TMDC-graphene superlattices at $a = 0, 1$ and 2 are given in Table S S5 and the in plane lattice constant for the bulk TMDCs are given in Table S S6. For the TMDC-graphene superlattices these are for the overall supercell and not transition metal to transition metal distances. These values for the superlattices are plotted in the main manuscript in Figures 2(c) and 2(d).

Table S4: The energetic costs of forming $Li_{a=1}MoS2C_b$ from LiC_6 and various other compounds, based upon equation S12, for the differing number of graphene layers (b = 3.375, 6.750 and 10.125 corresponding to 1, 2 and 3 layers of graphene).

	Lower Limit		Upper Limit	
b	Form. En. (eV)	β	Form. En. (eV)	β
3.375	-1.9736	0.2188	-2.5676	0.5
6.750	-1.5547	0.0	-2.6108	0.5
10.125	-1.6174	0.0	-2.6734	0.5

Table S5: In plane and out of plane lattice constants for TMDC-Graphene superlattices in Angstroms. These values are for the supercells considered, not the primitive unit cell values. The values of a correspond to the relevant levels of filling discussed in the main article.

	Lat. in plane (Å)			Lat. out of plane (Å)		
TMDC	a = 0	a = 1	a = 2	a = 0	a = 1	a = 2
MoS_2-H	12.7698	12.8818	13.0662	9.8817	10.4614	10.2790
MoS_2-T	12.8141	13.0206	13.1266	9.9824	10.3905	10.0901
WS_2-H	12.7725	12.8824	13.0562	9.8576	10.4230	10.2968
SnS_2-T	7.3890	N/A	7.4713	9.7767	N/A	11.3144
ScS_2-R	13.0551	13.1026	13.1521	9.5638	10.1178	9.8823
ScS_2-T	13.0551	13.0944	13.1145	9.5638	10.0900	10.07266
NiS_2-T	12.2687	12.3295	12.4748	8.9549	10.1941	9.8154
MnS_2-T	8.8761	8.9030	8.9978	9.4412	10.0015	9.8557
TiS_2-T	13.6970	13.7446	13.8187	9.5603	10.2224	10.0344

The supercells for $a = 1$ were constructed by taking the fully relaxed supercells for $a = 2$ and uniformly removing lithium. For the $a = 1$ structures where all the lithium was on one side of the TMDC layer this meant that all the lithium on one side was removed. For structures where the lithium is equally spread over both sides of the TMDC layer, lithium from every other lithium site. An example of this is shown in Figure SS1.

Table SS7 show the strain on the TMDC and graphene layers in the superlattices along with their respective formation energies. These were all calculated from graphene supercells of the same size as the superlattices and a single unit cell of the TMDCs, the only exception to this is MnS_2-T. Within the table, MnS_2 T* is the strain and formation energy as calculated with the above method and MnS_2-T uses a supercell of the TMDC layer instead. This was done as manganese's potential spin states are complex and numerous. To provide a direct comparison of the spin states, in this case we used the same supercell for the bulk TMDC

Table S6: The in plane lattice constant for the bulk TMDCs for differing levels of intercalation (none, $a = 0$ and partially filled, $a = 1$). The graphite supercell average carbon-carbon bond lengths in Angstroms.

TMDC	Lat. in plane (Å) a = 0	a = 1	Graphite (Å) C-C dist.
MoS_2-H	3.1615	3.1721	1.42407
MoS_2-T	3.1821	3.2822	1.42417
WS_2-H	3.1650	3.1492	1.42417
SnS_2-T	3.6707	3.7772	1.42418
ScS_2-R	3.7492	3.6234	1.42417
ScS_2-T	3.6291	3.6129	1.42417
NiS_2-T	3.3788	3.3945	1.42418
MnS_2-T	3.5713	3.3537	1.42424
TiS_2-T	3.4136	3.4279	1.42420

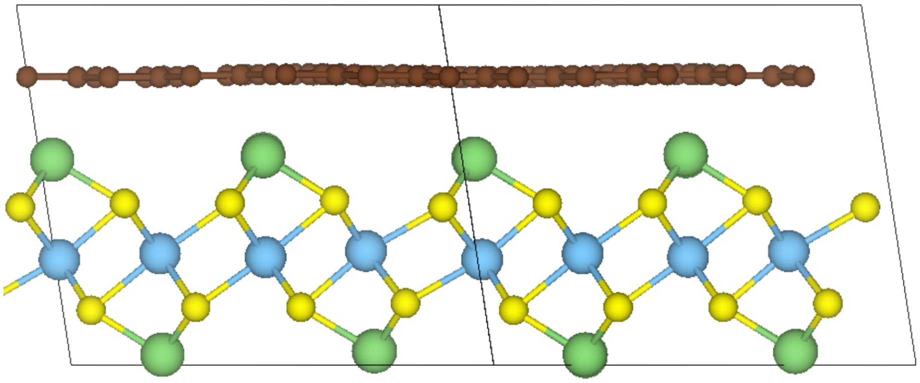

Figure S1: The structure of one of the TMDC-graphene superlattices for $a = 1$ where the lithium is spread over both sides of the TMDC.

and the superlattice.

Voltages

The voltages for the TMDC-graphene superlattices for $a = 0 \rightarrow 1$ and $a = 1 \rightarrow 2$ and the TMDCs without graphene for $a = 0 \rightarrow 1$ are given in Table S S8. These values for the TMDC-graphene superlattices are plotted in the main manuscript in Figure 2(a).

Table S7: The ratios of MX_2 to C along with the strain associated with each layer for the 9 supercells generated using the ARTEMIS[1] package and the formation energies per unit area. The strains are calculated for the TMDCs with no lithium compared with their superlattices with no lithium ($a = 0$).

TMDC	№ MX_2	C:MX_2 ratio (b)	Strain on TMDC (%)	Strain on Graphene (%)	Formation energy (meV/Å^2)
MoS_2 H	16	3.3750	0.9794	-0.3630	0.8610
MoS_2 T	16	3.3750	-0.5935	-0.0253	-8.9538
WS_2 H	16	3.3750	0.8873	-0.3536	0.04295
SnS_2 T	4	4.5000	0.6485	-0.1490	6.5286
ScS_2 R	13	4.3077	-3.4362	0.01825	13.3777
ScS_2 T	13	4.3077	-0.2399	0.01825	19.8208
NiS_2 T	13	3.8462	-3.4350	-0.5259	5.8030
MnS_2 T	7	3.7143	-4.7425	-0.2044	64.2991
MnS_2 T*	7	3.7143	-7.1114	-0.2044	-10.1196
TiS_2 T	16	3.8750	0.2909	-0.2700	11.6890

Volumetric expansion

The volumetric expansion the the TMDC-graphene superlattices for $a = 0 \rightarrow 1$ and $a = 1 \rightarrow 2$ and the TMDCs without graphene for $a = 0 \rightarrow 1$ are given in Table S S9. These values for the TMDC-graphene superlattices are plotted in the main manuscript in Figure 2(b).

Effect of additional Graphene

The voltages, E_{IS}, % volumetric expansion and % local expansion for MoS_2-T as the number of graphene layers are increased are given in Table S S10. These are calculated for lithium contents of $a = 0$ and $a = 1$ in $Li_aMoS_2C_b$ and are plotted in the main manuscript in Figure 4.

The local expansion for bulk MoS_2-T is the distance between the closest sulphur atoms in the two neighbouring TMDC layers, this effectively includes the change in the TMDC layer itself and the change in the van der Waals gap both above and below it as lithium is added. For the superlattices we have used the distance from the graphene layer above the TMDC to the one below, also capturing the change in the TMDC layer and the two van der

Table S8: Voltages for TMDC-Graphene superlattices and their respective TMDCs without graphene.

	Voltage (eV)			
	TMDC-Graphene		TMDC	% decrease
TMDC	a = 0 → 1	a = 1 → 2	a = 0 → 1	a = 0 → 1
MoS_2-H	0.4295	0.4085	0.7205	40.3830
MoS_2-T	1.5260	0.2905	1.7000	10.2324
WS_2-H	0.3971	0.07090	0.4029	1.4442
SnS_2-T	1.4470	1.4470	1.6373	11.6228
ScS_2-R	2.9596	1.1837	3.1332	5.5409
ScS_2-T	2.7294	1.0945	3.2064	14.8762
NiS_2-T	1.4581	1.3110	1.9911	26.7691
MnS_2-T	1.3812	1.0520	2.2565	38.7901
TiS_2-T	1.5092	1.0523	2.0571	26.6346

Table S9: Volumetric expansion for TMDC-Graphene superlattices and their respective TMDCs without graphene.

	Volumetric expansion (%)		
	TMDC-Graphene		TMDC
TMDC	a = 0 → 1	a = 0 → 2	a = 0 → 1
MoS_2-H	7.6632	8.9058	9.4792
MoS_2-T	6.4838	6.0682	9.6696
WS_2-H	6.6700	9.1470	8.5202
SnS_2-T	6.5654	18.3205	16.0291
ScS_2-R	6.5399	4.8702	14.5463
ScS_2-T	6.1368	6.2799	13.2120
NiS_2-T	14.9693	13.3228	24.9218
MnS_2-T	5.7221	7.2745	-2.8196
TiS_2-T	6.4534	6.8242	15.9876

Waals gaps.

Charge Analysis

A Bader charge analysis[2–5] was carried out on various MoS_2-T systems. Table S11 shows the Bader charges of the different species present in MoS_2-T with graphene as the concentration of carbon is increased without any lithium. Also included are the base TMDC without graphene and graphene without the TMDC.

Table S12 shows the Bader charges of the different species present in Li_aMoS_2-T with graphene as the concentration of carbon is increased with lithium at at $a = 1$. Also included

Table S10: The voltage, E_{IS}, % volumetric expansion and % local expansion for MoS_2-T as the number of graphene layers are increased.

	Carbon atoms per MX_2, b			
	0	3.375	6.750	10.125
Voltage	1.7000	1.5260	1.4475	1.4473
E_{IS}	-0.0248	-0.1906	-0.2548	-0.2341
% expansion	9.6696	6.4838	4.6312	3.7220
% Loc. exp.	4.5396	3.9652	4.1207	7.4130

Table S11: The average charge on the different species present in the MoS_2-T graphene superlattices without lithium as b is increased. $b = \infty$ is AA stacked graphite.

	№ electrons lost				
	C:MX_2 ratio (b)				No TMDC
Species	0	3.375	6.750	10.125	∞
Mo	1.7935	1.7439	1.7012	1.7033	N/A
S	-0.8967	-0.8855	-0.8752	-0.8778	N/A
C1	N/A	0.0080	0.0075	0.0067	N/A
C2	N/A	N/A	0.0071	0.0022	N/A
C3	N/A	N/A	N/A	0.0066	N/A
C Ave.	N/A	0.0080	0.0073	0.0052	0.0000

are the base TMDC without graphene and graphene without the TMDC and 2 Li-ions per 16 carbon atoms.

Additional MoS_2

We present the results of including an extra layer of MoS_2-T in these MoS_2-T - graphene superlattices, this is equivalent to $b = 1.6875$. However, there are multiple potential configurations for the Li within this system, and we have presented only one configuration (shown in Fig. S2 (d)). In this structure, the forces for this system are converged to ≈ 0.659 eV/Å (whereas in the main manuscript the forces are converged to 0.01 eV/Å). Figure S2 (a-c) the voltage and E_{IS} of the 2 layer MoS_2-T - 1 layer graphene structure is compared to the other MoS_2-T/Graphene supercells investigated. The voltage is ≈ 1.588 V and the E_{IS} is ≈ -0.141 eV, and as expected, these lie between the results for 1 layer of MoS_2-T with 1 layer of graphene and bulk MoS_2-T.

Table S12: The average charge on the different species present in the MoS_2-T graphene superlattices with lithium as b is increased. $b = \infty$ is AA stacked graphite with 2 Li to 16 C.

			№ electrons lost		
		$C:MX_2$ ratio (b)			No TMDC
Species	0	3.375	6.750	10.125	∞
Mo	1.6981	1.6198	1.6214	1.6221	N/A
S	-1.2823	-1.1476	-1.1447	-1.1421	N/A
C1	N/A	-0.0591	-0.0306	-0.0281	N/A
C2	N/A	N/A	-0.03092	-0.0070	N/A
C3	N/A	N/A	N/A	-0.0281	N/A
C Ave.	N/A	-0.0591	-0.0307	-0.0211	-0.1068
Li	0.8665	0.8746	0.8757	0.8757	0.8541

Diffusion barriers

For large supercells such as these, the number of unique diffusion pathways that one could consider is significantly larger than in a normal bulk unit cell. For example, in the MoS_2-graphene supercell, the number of Li sites (adjacent to the sulphur) is 32, meaning that one would need to consider 32 pathways for Li diffusion to just compare the diffusion between the two adjacent Li sites. When considering the larger cell, these pathways become significantly more complex. As a single case study, for the SnS_2-graphene system, which is the smallest of the systems considered, we have investigated one of the possible diffusion pathways. This pathway is shown in Figure S3, this pathway has a diffusion barrier of ≈ 0.257 eV. This is roughly half the diffusion barrier that was found for bulk SnS_2 (of ≈ 0.5 eV) found by Price *et al.*,[6] a reduction compared to the bulk is observed by others as well.[7] However this one diffusion pathway may not fully describe the full diffusion of Li through these systems and further study is warranted to explore this fully.

AIMD stability analysis

The systems considered in this article are large supercells consisting of 100's of atoms. For a full consideration of AIMD, one would normally want multiple picoseconds of simulation

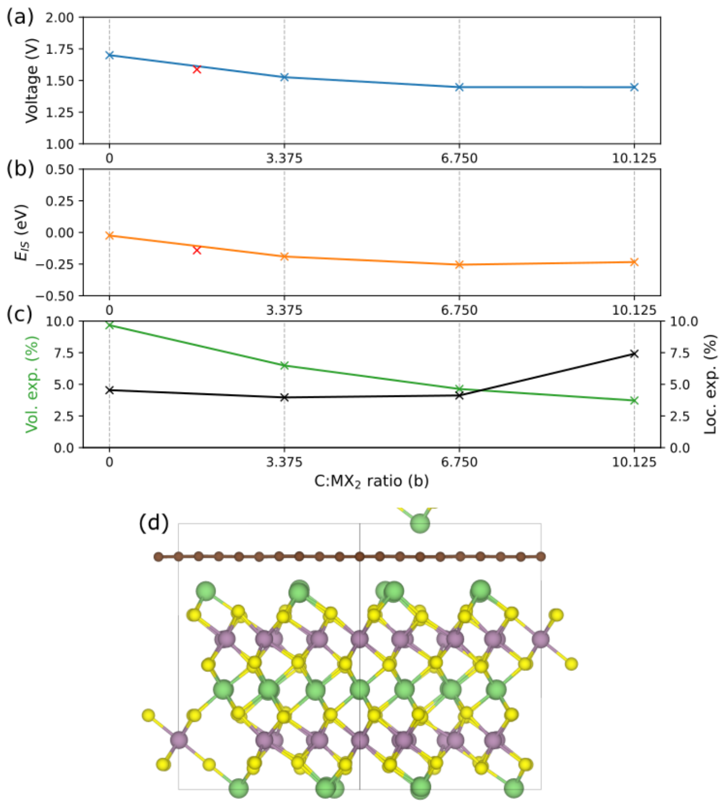

Figure S2: (a) The Open Circuit voltage and (b) E_{IS} for MoS_2-T as the number of layers of graphene is increased for a lithium content of $a = 0$ and $a = 1$. (c) shows both the total volumetric expansion in green and the local expansion in the z-axis in black. For the superlattices this is measured from the graphene layer below the TMDC to the graphene layer above, for the TMDC on its own we have used the distance between the closest sulphur atoms in the two neighbouring TMDC layers. The red crosses on the voltage and E_{IS} plots are for the 2 layer MoS_2-T - 1 layer graphene system and (d) shows the structure of this system.

to carry out a full stability analysis. For these systems, this remains unfeasible with current HPC resources. We can state here that the SnS_2-T - Graphene and $LiSnS_2$-T - Graphene systems are stable for approximately 0.1 ps, but this is vastly insufficient to state that these systems are truly phonon stable. However, in our previous works[6,8] we have shown the TMDCs individual phonon stability (without graphene) for select systems. As the interaction here is weak, we believe this would remain to be true, but a full phonon dynamics calculation (taking into account interplanar interactions) would be required.

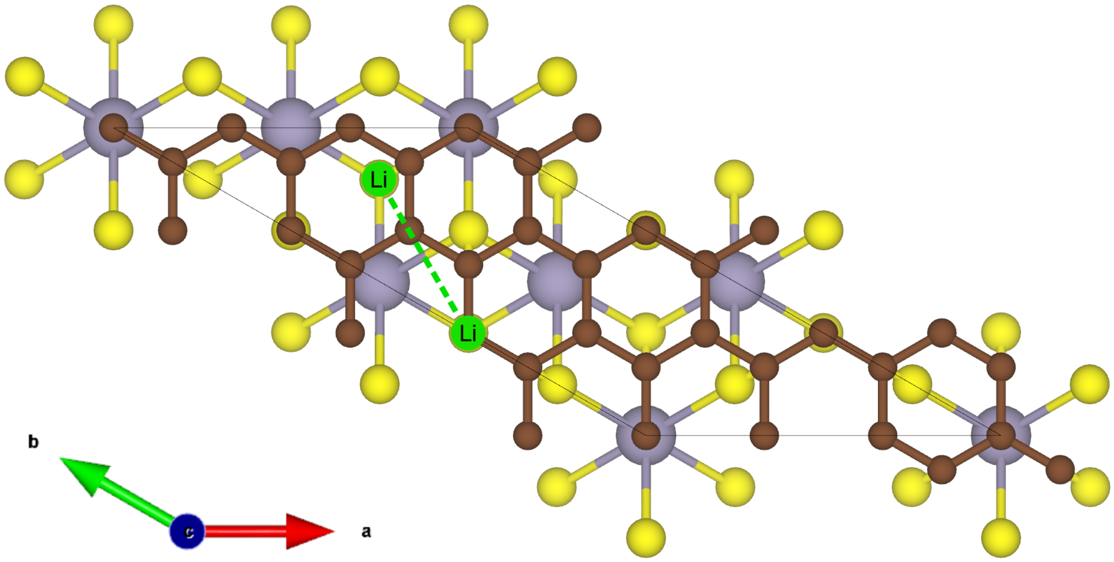

Figure S3: Schematic of the Li NEB pathway within the SnS_2-graphene superlattice.

References

(1) Taylor, N. T.; Davies, F. H.; Rudkin, I. E. M.; Price, C. J.; Chan, T. H.; Hepplestone, S. P. ARTEMIS: Ab initio restructuring tool enabling the modelling of interface structures. *Computer Physics Communications* **2020**, *257*, 107515.

(2) Tang, W.; Sanville, E.; Henkelman, G. A grid-based Bader analysis algorithm without lattice bias. *Journal of Physics: Condensed Matter* **2009**, *21*, 084204.

(3) Sanville, E.; Kenny, S. D.; Smith, R.; Henkelman, G. Improved grid-based algorithm for Bader Charge Allocation. *Journal of Computational Chemistry* **2007**, *28*, 899–908.

(4) Henkelman, G.; Arnaldsson, A.; Jónsson, H. A fast and robust algorithm for Bader decomposition of charge density. *Computational Materials Science* **2006**, *36*, 354–360.

(5) Yu, M.; Trinkle, D. R. Accurate and efficient algorithm for Bader Charge Integration. *The Journal of Chemical Physics* **2011**, *134*, 064111.

(6) Price, C. J.; Baker, E. A. D.; Hepplestone, S. P. First principles study of layered transi-

tion metal dichalcogenides for use as electrodes in Li-ion and Mg-ion batteries. *Journal of Materials Chemistry A* **2023**, *11*, 12354–12372.

(7) Yu, X.; Zhao, G.; Liu, C.; Wu, C.; Huang, H.; He, J.; Zhang, N. A MoS_2 and Graphene Alternately Stacking van der Waals Heterstructure for Li^+/Mg^{2+} Co-Intercalation. *Advanced Functional Materials* **2021**, *31*, 2103214.

(8) Price, C. J.; Pitfield, J.; Baker, E. A. D.; Hepplestone, S. P. First principles study of layered scandium disulfide for use as Li-ion and beyond-Li-ion batteries. *Physical Chemistry Chemical Physics* **2023**, *25*, 2167–2178.
